# Aurignacian dynamics in Southeastern Europe based on spatial analysis, sediment geochemistry, raw materials, lithic analysis, and use-wear from Românești-*Dumbrăvița*

**DOI:** 10.1038/s41598-022-15544-5

**Published:** 2022-08-19

**Authors:** Wei Chu, Scott McLin, Luisa Wöstehoff, Alexandru Ciornei, Jacopo Gennai, João Marreiros, Adrian Doboș

**Affiliations:** 1 Faculty of Archaeology, Leiden University, Einsteinweg 2, 2333 CC Leiden, The Netherlands; 2 Institute of Prehistoric Archeology, University of Cologne, Weyertal 125, 50923 Cologne, Germany; 3 Paleoanthropology, Senckenberg Center for Human Evolution and Paleoenvironment, Eberhard Karls Universität Tübingen, Tübingen, Germany; 4 Institute of Crop Science and Resource Conservation, Soil Science and Soil Ecology, University of Bonn, Bonn, Germany; 5 Department of Paleolithic Archaeology, Institute of Archaeology, “Vasile Parvan” of the Romanian Academy, 11 Henri Coanda Street, Sector 1, 010667 Bucharest, Romania; 6 Laboratory for Traceology and Controlled Experiments (TraCEr), MONREPOS – Archaeological Research Centre and Museum for Human Behavioural Evolution, RGZM, Mainz, Germany; 7 ICArEHB – Interdisciplinary Center for Archaeology and Evolution of Human Behaviour, University of Algarve, Faro, Portugal; 8 Institute for Prehistoric and Protohistoric Archeology, Johannes Gutenberg University, Mainz, Germany

**Keywords:** Archaeology, Geochemistry, Geomorphology

## Abstract

The Aurignacian is one of the first cultural-technological traditions commonly associated with the expansion of *Homo sapiens* in Europe. Early *Homo sapiens* demographics across the continent are therefore typically inferred using the distribution of Aurignacian assemblages. Western Romania has been used as a tie-point to connect the well-researched lithic assemblages from the eastern Mediterranean and Western Europe through its early *Homo sapiens* fossils. However, Romania’s archeological record remains underexplored thereby hindering our ability to directly connect better understood regions through time and space. Here we report on excavations from the open-air Middle/Upper Paleolithic site of Româneș﻿ti-*Dumbrăvița* I in southwestern Romania. Three stratified Paleolithic assemblages were extensively excavated within a 1-m-thick eolian-deposited sequence. Spatial, geochemical, raw material, techno-typological, and use-wear analysis of the site reveal patterns of artifact configuration, resource exploitation, fire history, knapping objectives, and functionality. Taken together, Românești-*Dumbrăvița* I is the first well-contextualized archeological site in close spatiotemporal proximity to many early, well-preserved human fossils and in East-Central Europe.

## Introduction

The dispersal of early modern humans into Europe is one of the main debates in paleoanthropology. A main hypothesis indicates that the Danube was a main axial migration route for modern humans to move into the upper catchment where behavioral developments subsequently took place and spread to other parts of Europe^1–5^. This scenario is intimated by early archaeological remains in the Upper and Lower Danube basins and corroborated by paleoanthropological remains and ancient mitochondrial DNA^6–8^. Still, a lack of robust archeological evidence from the middle of the continent makes it difficult to verify these models and understand how early *Homo sapiens* consolidated their presence across Europe^9,10^.

Western Romania is an important region for testing *Homo sapiens* dispersal hypotheses as it lies along the Danube Valley and astride some of the earliest known associated Aurignacian sites in Central Europe and the hypothesized Initial Upper Paleolithic progenitor industries in Moravia and Bulgaria^11–13^. It has additionally furnished some of the oldest undisputed *Homo sapiens* fossils in Europe^14–16^ along with associated fossil footprints and figurative cave art^17,18^.

**Figure 1:**
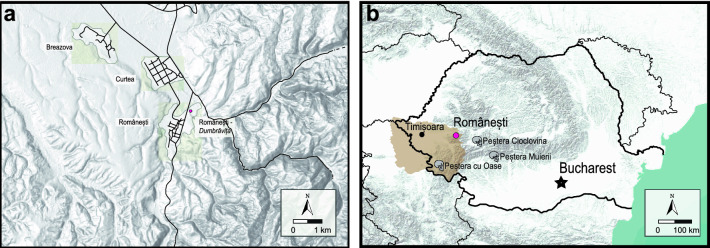
Position of Românești-*Dumbrăvița* (**a**) near the eponymous village in the Banat region; and (**b**) within Romania. The Banat is shaded in brown (Projection is latitude–longitude WGS84).

Still, Romania’s Aurignacian has been neglected in Pan-European discussions even though assemblages have been known for decades^19–21^. Of particular importance are the sites in Banat region that include Coșava-*Cuca*, Temerești-*Dealul Vinii,* and Românești-*Dumbrăvița* (hereafter Românești)^22,23^. Further afield but still in Banat are Tincova, Crvenka-At (Serbia), and Tabula Traiana Cave, sites that have additionally contributed to Aurignacian chronology and settlement patterns in East-Central Europe^24–31^. Banat sites are of particular importance because they are the closest contemporary sites to the early *Homo sapiens* findspots at the Peștera cu Oase, Peștera Muierilor, and Peștera Cioclovina (Fig. 1b)^14–16,32^.

Interpretations of Banat sites and their role in *Homo sapiens* dispersals have been hindered by unclear stratigraphic proveniences, few radiometric ages, small assemblage sizes, and a lack of contextual information. Even though Românești has been established as a key early Upper Paleolithic (eUP) site in East-Central Europe^33–37^, five main factors have limited Românești’s integration into transcontinental narratives:

1. Broadscale proveniencing, selective artifact recovery and/or the small-scale extent of past excavations have restricted inferring the degree and nature of post-depositional processes and anthropogenic spatial arrangements.
2. Abundant burned artifacts absent of combustion features have hindered appraisals of on-site anthropogenic fire activity to evaluate settlement dynamics.
3. Previous analyses of Banat Aurignacian assemblages have produced conflicting results limiting their integration within broader intra-/inter-regional eUP frameworks^26,34^.
4. Unknown raw material proveniences have impeded assessments of past regional landscape use and the site’s connection to wider mobility patterns.
5. The functions of eUP lithic forms remain uncertain limiting inferences of early European *Homo sapiens* subsistence strategies and behavior^38–41^.

This paper presents results of new excavations from Românești during 2016–19 describing site-level spatial investigations, sediment geochemistry, fire residue analysis, techno-typology, raw material economy, and use-wear to provide a detailed assessment of occupation and technological activities at an open-air Aurignacian site in a key part of Europe. The results provide the only cultural link to the early, well-preserved fossils from a region in Europe that is otherwise devoid of secure archaeological sites.

## Research background

The site of Românești is situated on a river terrace outside Românești village near the confluence of the Bega Luncani and Bega Poieni rivers in Banat of Romania (Fig. 1a). The site comprises two localities, Românești I and II (80 m apart) excavated between 1960–1964 and 1967–1972^22^.

Românești I was excavated to an average depth of 1.3 m across 450 m^2^ (Supplementary Fig. 1). Six Paleolithic levels were reported though they were never found in full succession. The lowest level (I) was attributed to a “Quartzite Paleolithic”, being manufactured exclusively on Quartzite, and typified by Mousterian points, naturally backed knives, and Upper Paleolithic forms (prismatic cores and endscrapers). The subsequent four levels (II–V) were attributed to the Banat Aurignacian. Level VI was typified by Aurignacian elements alongside later Upper Paleolithic artifacts such as backed bladelets, Gravette points and scalene triangles. No faunal remains or features were recovered from the site.

Research resumed following the discovery of the antiquity of the *Homo sapiens* fossils in Banat^14,42^ and the re-examination of the nearby Tincova assemblage^43,44^. Test pits were installed at Românești in 2009–2010 to validate the stratigraphy, evaluate the frequency of smaller artifacts, obtain absolute ages, and frame the site within a wider European context^34^. Radiometric dates from optically stimulated luminescence (OSL) and thermoluminescence (TL) bracketed the Aurignacian artifacts to between 42.1 and 39.1 ka ago^35^.

## Results

### Spatial analysis

**Figure 2:**
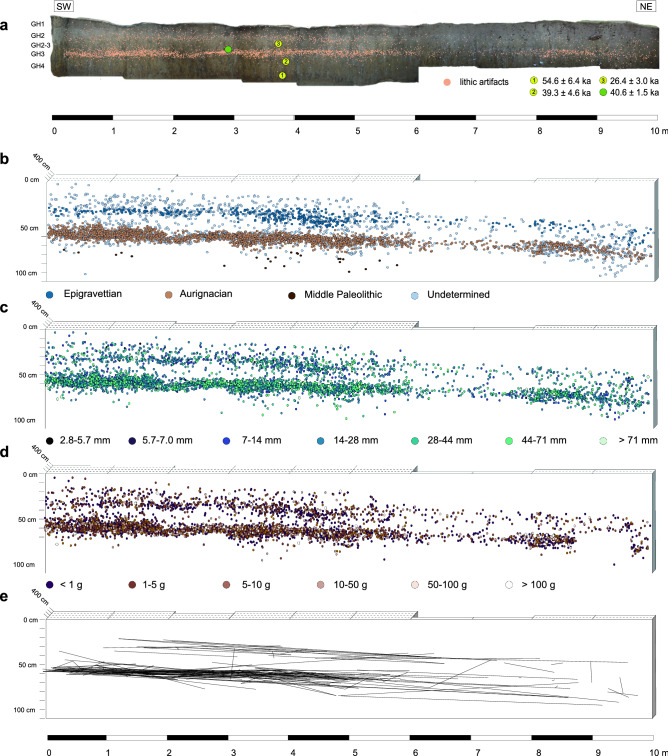
Horizontal projection of all piece-provenienced lithic artifacts of the 2016–2019 excavations at Românești-*Dumbrăvița* I. (**a**) composite orthophoto of the northwestern profile. Yellow circles represent positions of OSL dates and green circle represents composite TL dates^35^; (**b**) archeological layers; (**c**) artifact distribution according to maximum length; (**d**) artifact distribution according to weight; (**e**) lithic refits. Top scales in **b**–**e** indicates horizontal depth of excavation.

**Figure 3:**
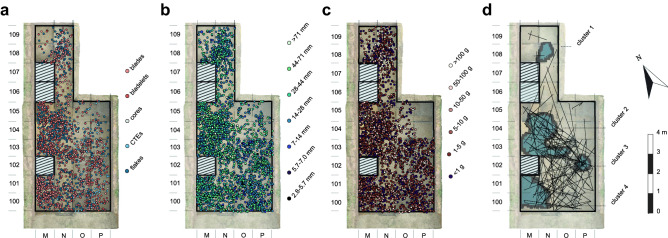
Top-down orthophoto (final excavation depths) of the 2016–19 excavations at Românești-*Dumbrăvița* I with lithic artifacts projected from GH3 (Aurignacian). (**a**) Lithic type distribution; (**b**) lithic size distribution; (**c**) lithic weight distribution; (**d**) lithic refits and scan test results highlighting lithic clusters (blue; background is transparent to facilitate viewing of refit lines).

5655 piece-provenienced artifacts were recovered in three discrete find layers associated with the Epigravettian, Aurignacian, and Middle Paleolithic (Fig. 2). For the Aurignacian layer in Geological Horizon (GH) 3 (see “Methods”), lithics show no visible trend in size sorting according to length and weight (Figs. 2, 3).

For GH3, a density plot showed an inhomogeneous spatial distribution of artifacts. A chi-squared (χ^2^) test indicated that for most excavation units, the number of lithics exceeded the range of expected values of a random pattern with the highest deviation in unit P102 (R = 15) confirming an inhomogeneous distribution (χ^2^ (0, N = 3737) = 1216.5, *p* < 0.01; Supplementary Fig. 2). A K function demonstrated that artifacts were clustered at short distances (*r* < 50 cm) and were randomly distributed thereafter (Supplementary Fig. 3). A scan test uncovered four clusters (CL1–4) with radii ranging between 17 and 40 cm (Fig. 3d). The relative probabilities showed substantial associations between bladelets and cores in CL1, cores and core trimming elements in CL3, and blades and bladelets in CL4 (Supplementary Fig. 2).

A nearest neighbor equality function demonstrated a positive spatial correlation in the distribution between bladelets–cores and partially between blades–bladelets (Supplementary Fig. 3) indicating that for example, between bladelets and cores, bladelets tend to be closer to other bladelets whereas cores tend to be closer to other cores. In the case of blades and bladelets, a positive correlation occurred only at short distances (between 3 and 10 neighboring artifacts). Similarly, blades and bladelets were positively associated but only after six neighboring artifacts.

### Black carbon identification

**Figure 4:**
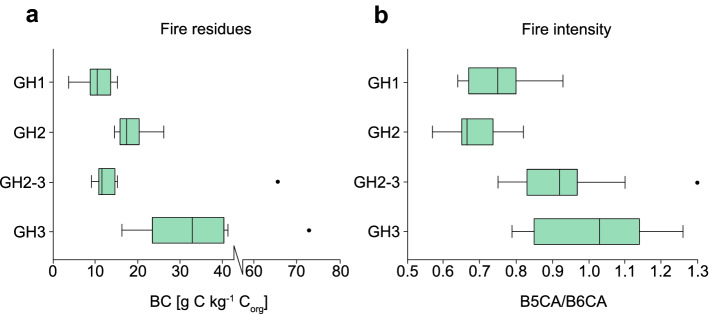
Black carbon analyses from Românești-*Dumbrăvița* I. (**a**) Black carbon contents as determined by BPCA analysis in the four geological horizons; (**b**) black carbon quality as indicated by B5CA/B6CA ratios in the four geological horizons. Increasing values represent low temperature fires, a shift to lower values points to higher combustion temperatures.

Fire residue input, i.e., black carbon (BC) content, was found in all samples with amounts ranging from 3.7 ± 1.2 to 72.6 ± 7.0 g C kg^−1^ C_org_. The lowest BC contents were detected in GH1 with an average of 10.7 ± 2.1 g C kg^−1^ C_org_, followed by GH2-3 with 16.8 ± 3.1 g C kg^−1^ C_org_ (Fig. 4a). Both GH2 and GH3, with dense accumulations of lithics, showed increased BC contents (18.4 ± 3.5 and 33.0 ± 5.7 g C kg^−1^ C_org_, respectively). GH2-3 and GH3 each had one outlier with high BC contents (GH2-3: 65.6 ± 11.3 g C kg^−1^ C_org_; GH3: 72.6 ± 7 g C kg^−1^ C_org_). Fluctuations in BC were low within horizons (GH1, GH2 and GH2-3) apart from GH3, which showed comparatively high heterogeneity in BC values across the profile.

Benzene polycarboxylic acids (BCPA) composition ideally allows the reconstruction of past fire regimes and changes in fire temperature and is expressed by the ratio of pentacarboxylic acid to mellitic acid (B5CA/B6CA)^45^. The lowest B5CA/B6CA ratio values were found in GH2 with an average of 0.7 ± 0.1, followed by GH1 and GH2-3 (0.8 ± 0.1 and 0.9 ± 0.1); GH3 had the highest ratio with 1.0 ± 0.1 (Fig. 4b).

### Raw materials and MAN analysis

The GH3 lithic assemblage is composed of local shear zone related cherts supplied from locations close to faults, local gravels, and creeks as well as quartzite and other hard metamorphic rocks from the nearby gravels. Non-local raw materials notably “black flint” (non-local fault chert) and greyish-bluish chert (other siliceous rocks; Supplementary Fig. 4) are also present.

**Figure 5:**
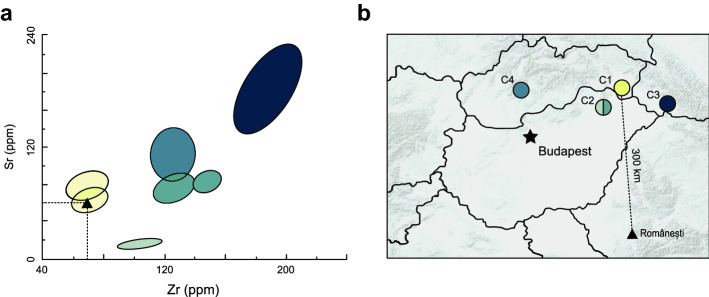
Source of the obsidian artifact from Românești-*Dumbrăvița* I, GH3. (**a**) Bivariate plot of 90% confidence intervals of Zr and Sr concentrations of Carpathian obsidian sources^47^ and the single obsidian artifact from Românești; (**b**) map of Carpathian obsidian sources and the site of Românești.

Six main raw material categories were distinguished based on macroscopic criteria and thin sections (Supplementary Fig. 4)^46^: Local shear zone related cherts found in the surveyed area (Poieni-Pietroasa fault chert, Căldărilor fault chert, ductile shear zone related cherts), metamorphic hard rocks found in the surveyed area (quartzite, vein quartz, graphitic quartz), non-local fault cherts, diagenetic cherts (very fine-grained diagenetic cherts, bioclastic cherts, non-skeletal cherts, siliceous mudstone/marlstone), obsidian and other siliceous rocks, indeterminate cherts, and (partially) burned rocks. Sr and Zr from an obsidian blade found in GH3 matches the obsidian outcrops of the C1b cluster from Cejkov, Slovakia 300 km away but could also fit within the C1a cluster 7 km away in Vinicky, Slovakia (Fig. 5)^47^.

2942 lithics were used in a Minimum Analytical Nodules Analysis (MANA) as the lithologies of some small, burned, or indeterminate raw materials could not be confidently assigned to a Minimum Analytical Nodule (MAN; Supplementary Fig. 4). Artifacts were grouped into 194 MANs (Supplementary Fig. 4), further characterized by the number of artifacts contained in a group (Supplementary Fig. 5): single item MANs (1 artifact) and multiple item MANs (two to tens of artifacts). The non-local raw materials from the assemblage are represented by single item MANs or multiple item MANs (< 10 artifacts) introduced as end-products and preformed cores and a few multiple item MANs (> 40 artifacts) introduced as preformed cores supplied from distances ranging from 13–60 km. MANs were assigned to archeological layers based on the GH of most of the artifacts in a MAN (some contained artifacts from other GHs) and/or the presence of culturally specific tool types. The MANs were also confirmed by artifact refits in the same group that were performed during categorization (Figs. 2e,3d). Single item MANs from both assemblages contained tools, debitage, or in some isolated cases, knapping waste products, suggesting that other artifacts of that MAN were outside of the excavation area.

### Techno-typological analysis

**Figure 6:**
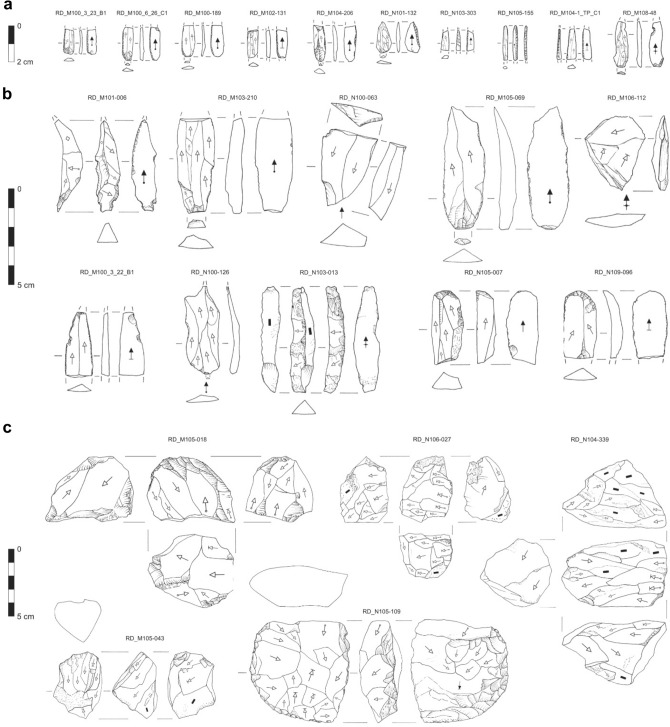
Selection of (**a**) bladelets, (**b**) blades and flakes and (**c**) (pre-) cores from Românești-*Dumbrăvița* I, GH3, 2016–19 excavations.

Cores primarily produced blades and bladelets or just bladelets on narrow longitudinal faces with frontal knapping (*narrow-fronted*) or on broad longitudinal faces with semi-circumferential knapping (*semi-rotating;* Fig. 6c; Supplementary Table 1). Narrow-fronted cores were prevalently initiated on large flakes, while semi-rotating cores were manufactured on nodules or cobbles. 50% of cores preserved cortex (Supplementary Table 1). Semi-rotating cores preserved cortex on the posterior faces while narrow-fronted cores preserved cortex on the lateral faces. Cores primarily had single and plain unipolar striking platforms where overhang was frequently abraded and knapping angles were moderately acute to acute.

In GH3, debitage is equally divided between flakes and blades/bladelets (Table 1). Debitage platforms were mainly plain, linear, or punctiform (Supplementary Table 2). Bulbs were mostly diffused among blades and bladelets, while flakes were diffused and pronounced. Overhang abrasion was ubiquitous, but dominant among blade and bladelet blanks. Lipping was infrequent though well represented among blade blanks.

**Table 1:** Classification of the piece-provenienced debitage from Românești-*Dumbrăvița* I, GH3.

	Blades (%)	Bladelets (%)	Flakes (%)	Total (N)
Complete	131 (21)	106 (21)	481 (68)	718
Proximal fragment	172 (28)	79 (16)	98 (14)	349
Meso-proximal fragment	84 (14)	86 (17)	45 (6)	215
Mesial fragment	96 (16)	87 (17)	19 (3)	202
Meso-distal fragment	48 (8)	95 (19)	18 (3)	161
Distal fragment	83 (13)	51 (10)	23 (3)	157
Fragment	2 (< 1)	1 (< 1)	26 (4)	29
Total debitage products	616 (34)	505 (28)	710 (39)	1831
Total Complete and Semi-Complete	263 (24)	287 (26)	544 (50)	1094

Most debitage was non-cortical, especially among blades and bladelet blanks. Extensive patches of cortex were only on initialization flakes. Cortical debitage was primarily semi-cortical found on lateral and distal positions. Negatives, preserving at least undisturbed width, were present on 35% of the debitage and were unidirectional; most showed a final bladelet removal.

Blade and bladelet profiles were primarily straight or twisted (Supplementary Table 3). Twisted profiles were found among asymmetrical blades and bladelets (Fig. 6a,b). Curved profiles were more frequent among blade blanks than among bladelet blanks. Complete and semi-complete debitage were mostly blanks or related to core shaping (Supplementary Table 4). Management debitage was more frequent among flakes and blades than bladelets (Supplementary Fig. 6).

72 artifacts were retouched (Table 2). Burins (dihedral or on truncations) were made on all debitage forms. Endscrapers were rare, manufactured on blades and a single flake. Retouched artifacts were primarily laterally retouched blades and bladelets, Dufour (subtype dufour) bladelets, and laterally backed blades and bladelets.

**Table 2:** Classification of retouched blade, bladelet and flakes from Românești-*Dumbrăvița* I, GH3.

	Blade					Bladelet					Flake						Total
	Asymmetric (%)	Maintenance (%)	Overshot (%)	Blank (%)	Total	Asymmetric (%)	Burin Spall (%)	Maintenance (%)	Blank (%)	Total	Core tablet (%)	Cortical (%)	Maintenance (%)	Blank (%)	Surface cleaning (%)	Total	
Burin on truncation	0	0	0	1 (7)	1 (5)	0	0		0	0	0	0	0	0	1 (20)	1 (7)	2 (3)
Dihedral burin	2 (100)	2 (67)	1 (100)	2 (14)	7 (35)	0	0	2 (100)	2 (7)	4 (11)	1 (100)	0	0	1 (20)	1 (20)	3 (21)	14 (20)
Endscraper	0	0	0	3 (21)	3 (15)	0	0	0	0	0	0	1 (50)	0	0	0	1 (7)	4 (6)
Dufour bladelet	0	0	0	0	0	1 (25)	0	0	10 (34)	11 (29)	0	0	0	0	0	0	11 (15)
Laterally backed	0	0	0	3 (21)	3 (15)	1 (25)	1 (33)	0	3 (10)	5 (13)	0	0	0	0	0	0	8 (11)
Laterally retouched	0	0	0	4 (28)	4 (20)	2 (50)	2 (67)	0	14 (48)	18 (47)	0	0	0	0	0	0	22 (31)
Retouched flake	0	0	0	0	0	0	0	0	0	0	0	1 (50)	1 (100)	4 (80)	3 (60)	9 (64)	9 (13)
Retouched fragment	0	1 (33)	0	1 (7)	2 (10)	0	0	0	0	0	0	0	0	0	0	0	2 (3)
Total	2	3	1	14	20	4	3	2	29	38	1	2	1	5	5	14	72

### Lithic use-wear analysis

Lithics showed sporadic use primarily on larger (> 5 cm) unretouched artifacts and evidence for two hafted armature points (Supplementary Fig. 7). While some lithics recovered from GH1 showed post-depositional modification^48^, lithics from GH3 retained fresh, unpolished surfaces. Of the 209 lithics analyzed, 167 (80%) showed no evidence of use-wear.

**Figure 7:**
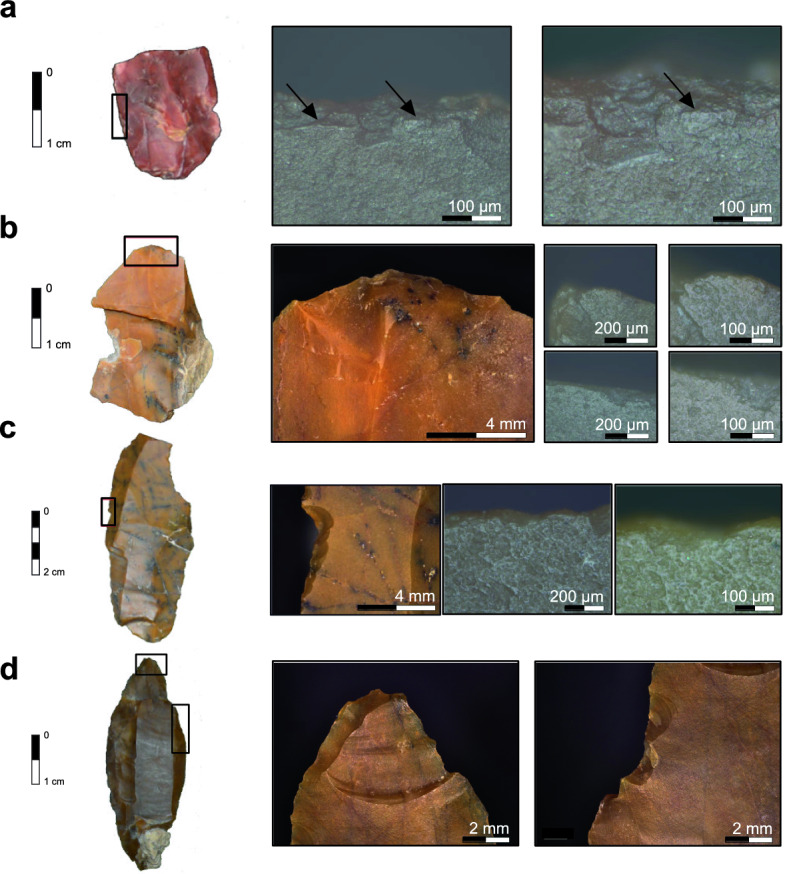
Use-wear traces found on lithic artifacts from Românești-*Dumbrăvița* I, GH3. (**a**) Localized microfracturing and compact polish of the lateral edge characteristic of hard animal material scraping (on the right side, 10 × and 20 × objective); (**b**) Abrasive polish and rounding of the distal end of the ventral surface characteristic for hide-scraping (on the right side, 10 × and 20 × objective); (**c**) Striated and extensive polish on the ventral surface of a steeply notched lateral edge characteristic of wood scraping (on the right side, 10 × and 20 × objective); (**d**) Macrofracture traces found on a lithic artifact. (**left**) spin-off fracture initiated on the dorsal distal tip on the lateral side with feather termination. (**right**) lateral ventral notching and edge scarring.

43 artifacts from GH3 showed localized use-wear traces (Fig. 7). Of these, 16 had poorly developed, extended generic polishes with features unattributable to a specific use. Of those that were diagnostic, eight exhibited small alternate flake scars on dorsal and ventral surfaces and nonstriated polishes indicative of vegetal cutting. Striated polishes from hide-scraping were observed among six while five exhibited nonstriated, ventral wood cutting polish. Five lithics were related to bone scraping and one was associated with antler processing.

Two laminar points had abrupt, isolated, proximal tip damage. One bore a feather-terminating ‘spin-off fracture’ (Fig. 6d). Abrupt localized flaking without polish was found on corresponding lateral edge locations suggesting that the artifact was hafted during use^49–51^.

Only one Dufour bladelet showed use-wear as an unspecific, nonstriated polish between retouch along the proximate lateral edge. The two manuports showed a single concave groove (c. 1 cm width; 0.5 cm depth) traversing the longitudinal center of a single face (Supplementary Fig. 8). No pitting or percussion marks were observed, and micrographs showed well-developed unidirectional striations and polish possibly consistent with wood polishing. Other areas of the artifact did not exhibit signs of use.

## Discussion

### Spatial analysis of artifacts

The vertical distributions of artifact weight and size in GH3 show no indications of post-depositional movement. As artifact weight distribution from GH3 at Românești shows a greater abundance of lighter lithics (50% for < 1 g) and shorter lithics (50% for 14–28 mm) upslope, downslope movements did not substantially alter the artifact distribution^52,53^.

Rather, the χ^2^ test and Ripley’s K function demonstrate anthropogenic aspects of the horizontal artifact distribution of GH3. Notably, blades occurred in all four clusters, whereas cores only substantially occurred in CL1 and core trimming elements only substantially occur in CL3 (Fig. 3). The dominance of blades, bladelets, and cores in CL1 and CL3 suggests that these clusters represent knapping areas of similar activities.

Lithic distributions and associations revealed by the nearest neighbor equality function suggest three different anthropogenic patterns. The first pattern relates to the correlated distribution of bladelets, blades, and cores; the second indicates an independent distribution of core trimming elements; the third relates to the change in the distribution of blades at short distances to a separate, different distribution at larger scales.

### Black carbon identification

BC in sediment samples were highest in GH3 (Fig. 4a). Overall, BC did not change with depth, thus the profile likely reflects past differences in BC accumulation rather than post-depositional processes. Sediment leaching was unlikely as water solubility of BC is generally low^54^. Nevertheless, bioturbation could have contributed to BC content heterogeneity in individual GHs^55–57^.

BC at Româneşti was highest in GHs with the highest artifacts concentrations coinciding with human occupation intensity (GH2 and GH3). This is supported by the lower BC contents in the horizons on top and between two find layers which had few lithics (GH1 and GH2-3, respectively; Fig. 4a). However, one sample in GH2-3 showed a comparatively high BC value given the overall low contents within that horizon. As GH2-3 had low artifact density, natural processes were likely the cause as sedimentary cracks may have caused surface water and BC to percolate downwards. In contrast, the distinct BC peak in GH3 where cracks were not observed, suggests an anthropogenic influence.

The increased fire activity in GH2 (indicated by BC content) was accompanied by high fire intensity (i.e., low B5CA/B6CA ratios; Fig. 4b) typical for domestic fires. Wood-fueled anthropogenic fires typically burn substantially hotter than natural fires (e.g., forest ground/grass fires)^45,58^. Compared to GH2, values for GH3 indicate lower fire temperatures. In GH3, overall artifact richness, increased BC contents, and ratio values could be interpreted as a mixed signal from both natural and domestic fires. However, it could also illustrate a change in fuel source induced by changing environmental conditions or anthropogenic fire behavior.

Fire frequency in grass-dominated environments is typically higher than in dense forests^59,60^. During the Late Pleistocene, grassland may have been the predominant vegetation type in Banat^61^, with potential temporary shifts to forest steppes during MIS 3^62^. At Româneşti, higher fire activity could explain the absence of macro-charcoal in the profile as it may have been reburned^63^. However, the near absence of organic residues at Româneşti and other regional open-air sites suggests that taphonomic processes may also be responsible.

### Raw materials and MAN analysis

The GH3 assemblage is composed of many multiple items MANs of local raw materials introduced to the site as slightly prepared, tested, or unprepared blocks from sub-autochthonous sources and nearby gravels. The modes of introduction, quantities, and distances from the sources indicate that non-local raw materials were acquired by direct procurement indicating a complex provisioning strategy^46^. The obsidian artifact from GH3 was introduced as an isolated end-product that may have been acquired by indirect procurement (exchange) from sites between Românești and Southeastern Slovakia.

Still, raw materials from Românești overall suggest a narrow raw material procurement territory (< 60 km) for the GH3 assemblage primarily supplied from nearby sources (< 10 km). Altogether, local and presumed non-local shear zone related cherts comprise 84% (90% including burned artifacts) of the GH3 assemblage. The same raw materials from Românești were used at other Aurignacian sites from central and northeastern Banat, but little is known about their modes of introduction and exploitation^23,30^. The MAN data suggest nuanced lithic procurement patterns at Românești: non-local raw materials track the incoming path of Upper Paleolithic hunter-gatherers and outline the scale of group mobility; the raw materials supplied from local sources are potentially connected to the site position and the activities carried out there.

### Techno-typology of GH3

GH3 shows on-site knapping aimed at bladelet production attested to by cortical flakes and shaped crests. Cores have single platforms where the flaking surface was installed on a narrow-fronted or broad (semi-rotating) longitudinal face. Though semi-rotating cores are fewer, their larger volume likely yielded comparable amounts of debitage. Narrow-fronted cores usually have one lateral cortical face attesting to their initiation on large decortication flakes. However, at least one pre-core (Fig. 6c; RD_N105-109) indicates that raw materials were also shaped to produce a crest on naturally narrow surfaces. Core platforms are unfaceted corresponding with debitage platforms and tablet dorsal faces. Assemblage knapping angles are mostly acute and striking platform overhangs are frequently abraded, both characteristic of laminar volumetric knapping by direct marginal percussion introduced with the Protoaurignacian^64^. Core faces tend to fuse into a single, broad, semicircular flaking surface throughout the reduction sequence. As narrow-fronted cores already possessed a narrow, straight flaking surface, it was sufficient to exploit the longitudinal ridges offered by the intersection of the two lateral core faces.

The GH3 assemblage shares several similarities with other contemporaneous eUP assemblages in East-Central Europe namely Kozarnika layer VII^65^, Siuren I layers H and G^66–68^, Berehove I AH I^69,70^ and Willendorf II AH 3 in Central Europe^12,71^. Preferentially retouched bladelets and microbladelets are main components of these inventories. All were manufactured from independent cores using broad, narrow flaking surfaces with semi-circumferential and sub-prismatic morphologies. Most bladelet profiles show flaking surfaces are organized to isolate a central, straight/slightly curved area achieved by knapping the lateral periphery of the flaking surface with off-axis, plunging laminar blanks, or by limiting the main flaking surface through lateral flakes. Striking platforms are usually plain and negatives are unidirectional. As such, these assemblages are recognized as Protoaurignacian, except Willendorf II AH 3, which is ascribed to the Early Aurignacian. The GH3 assemblage can therefore be attributed to the Protoaurignacian^37^.

Nevertheless, it is unclear to what degree Western European Aurignacian subcategories can be applied to those further east. Disjointed blade/bladelet production and microbladelets, aspects of the Early Aurignacian, are observed in many Southeastern Protoaurignacian assemblages^34,66,68,70^. This may be because carinated (Early Aurignacian) or prismatic (Protoaurignacian) cores classification can be subjective as they may exhibit similar reduction sequences^67,72,73^. Therefore, it is challenging to discern the Protoaurignacian from the Early Aurignacian in Central-Southeastern European record either due to the paucity of data or a genuine absence of subphases^12,36,74^. Nonetheless, the GH3 techno-typological characteristics and radiometric ages match eUP sites in the region currently ascribed to the Protoaurignacian.

### Lithic use-wear analysis

Most analyzed artifacts at Românești showed no resharpening, abrasion, rounding, or striations and were therefore unused or only used for short durations on softer materials. The low proportion of retouched artifacts across the assemblage suggests that resharpening was not a main factor in obscuring use-wear and that lithics were generally used ad hoc. The multiple (semi-)complete lithic reduction sequences found in GH3 suggests that lithics were therefore primarily abandoned byproducts characteristic of a workshop.

Among artifacts with use-wear, evidence was primarily generic and disassociated with specific artifact types or retouched edges implying that lithics were unsystematically exploited, perhaps repeatedly for a range of tasks. Domestic tools such as endscrapers, often related to discrete activities, are virtually absent from the site suggesting a functional orientation and focus of planning depth different from other Aurignacian sites^40,41,75^ at a time when domestic versus hunting tools were becoming increasingly differentiated^76^.

When attributable to a specific use, cutting and scraping wear show that vegetal and wood processing was the main on-site activity though a range of animal processing activities were also observed. Harder materials (i.e., wood, bone) are less represented than softer materials (i.e., hide, vegetal) suggesting that overprinting of prior use traces does not obscure the overall pattern.

Spin-off fractures on elongated blanks like those found at Românești are often cited as a diagnostic impact fractures (DIFs) indicative of projectile weaponry possibly delivered by composite tools^49,77–80^. However, DIFs can also infrequently occur because of trampling and other natural processes^77,81–84^. Even combined with the potential hafting traces on a single piece, it is tempting to ascribe the Românești artifacts with DIFs to projectile armatures though such near isolated evidence cannot robustly confirm evidence for projectile damage.

Still, the virtual absence of use-wear on Dufour bladelets may provide insight into projectile manufacture processes. Upper Paleolithic bladelets, particularly Dufour bladelets^38,85–87^ have long been associated with armature inserts or points^88–91^. The scarcity of use traces on the Românești Dufour bladelets indicate that they were either unmanipulated in substantial processing tasks or that use-wear traces were later removed by retouch. Here, the lack of bifacial polish on the unifacially retouched lateral edges would demonstrate that if used, they were not used for substantial lengths on hard materials.

This absence of use-wear would be consistent with them either being discarded before intended use or their use as hafted inserts or points. If used as inserts, weak generic polish from the armature, hafting material or even mastic residues would be expected on a lateral edge none of which would be observed. Rather, the absence of polish and intense alternating retouch of the Dufour bladelets may be the result of inserting the bladelet into the tip of the shaft and blunting/fitting/hafting the bladelet while rotating the shaft.

The proximity, comparable sizes, coarse-grained lithology, and dimensions of the two manuports with heavily polished rectilinear grooves implicate similar functions. Both grooves appear to be furrowed, perhaps in tandem, through intense polishing of branches and therefore may have been polishers/straighteners for wooden shafts. These artifacts bear resemblance to standardized forms of shaft-straighteners known from later periods, but which have also been recovered at other eUP sites^92–96^.

## Conclusion

A significant feature that has emerged from Aurignacian research is the potential for multiple chronologically, spatially, and technologically distinct *Homo sapiens* expansions across Europe^68,97–99^. Validating Trans-European dispersal scenarios, however, has remained difficult due to the bias towards Western European datasets. The new excavations from Româneşti contribute to our understanding of the earliest *Homo sapiens* in Europe by delivering the largest, well-contextualized Aurignacian lithic assemblage in East-Central Europe within a multi-layered site.

Taken together, Româneşti demonstrates that the onset of the East-Central European Upper Paleolithic, the Aurignacian marks a profound shift towards a large, spatially structured open-air site complex indicative of a sustained occupation or repeated visits^100^. This pattern, corroborated by neighboring Banat sites^21,23,29,30,33^, stands in contrast to ephemeral Late Middle Paleolithic assemblages^101–103^. Coupled with the contemporaneous increased anthropogenic fire input, GH3 implicates settlement dynamics at odds with a Southeastern European “pioneering phase” of rapid, unidirectional, westward expansion posited by iterations of the Danube Corridor Hypothesis^74,104^.

The use of good-quality local, meso-local, and exotic raw materials at the onset of the Aurignacian also demonstrates that Româneşti and the other Banat sites were repeatedly returned to from a range of distances up to 300 km and were persistent places in the landscape. Curated obsidian artifacts indicates that throughout the eUP, Româneşti was repeatedly returned to over a range of distances.

Româneşti’s Aurignacian assemblage also enables insights into the objectives and use of lithics. The narrow focus on bladelet production through a limited array of methods indicates that the site was continually geared towards a prescribed set of technological tasks. The near absence of domestic tools and the low frequency of use-wear almost exclusively on blanks, indicates that tools were generally used extemporaneously and that finished forms were exported. If the association of the site with armature production is valid, one might begin to envisage a retooling locale from which retouched bladelets were attached to armatures and transported offsite^105^.

The results of the large lithic assemblages and their high-quality contexts from the new excavations at Româneşti indicate profound changes in hominin occupation patterns at the onset of the early Upper Paleolithic from ephemeral to more persistent in relation to landscape dynamics during the Late Pleistocene in East-Central Europe. These data contribute to a clearer understanding of how early *Homo sapiens* mobility and how they consolidated their presence in Europe. Româneşti provides an important material culture context to the early European *Homo sapiens* fossil record delivering a key inflection point for continental-wide Aurignacian sites.

## Methods

### Excavations

In 2016, 17 m^2^ were excavated adjacent to the 2009 test-trench to provide correlation with previous radiometric dates and geochemical proxies (Supplementary Fig. 1). In 2018, the excavation was extended 8 m^2^ to the east and in 2019, it was expanded 4 m^2^ north and units M–P, 100–104 were excavated further until 20 cm of sterile sediments were reached. We conformed to the stratigraphic descriptions of Sitlivy et al.^37^ which used a four-part division of GH based on sedimentary color and composition (GH1–4) that coincided with lithic technological changes. However, as the transition between GH2 and GH3 were not always clear due to sedimentary interfingering, an intermediary level GH2-3 was designated when attributions were ambiguous.

The excavation surface was gridded in square meters that were subdivided into quadrants (A–D). Quadrants were excavated by removing sediment in 2 cm intervals. Archeological remains ≥ 5 mm in maximum dimension were spatially recorded in three-dimensions by measuring their center point with a Leica TS06 total station using EDMwin mobile software^106^. Excavated sediments were wet sieved in 5 mm and selectively in 2 mm mesh to facilitate finer artifact recovery.

Finds were predominantly chipped stone artifacts and their associated debris though larger allochthonous stones were also recovered. Organic preservation was modest: Only two fragments (2 cm) of poorly preserved conjoining dental enamel (possibly *Mammuthus sp.*) were recovered that did not yield collagen for dating. Sediment analysis by the Cologne Archeobotanical Laboratory did not recover microfloral remains from the profile.

### Spatial analysis

Spatial analysis was performed to clarify archeological stratigraphy, investigate post-depositional processes, and identify potential anthropogenic patterns. To ascribe lithics to archeological layers, lithic artifacts that vertically deviated from two neighboring artifacts by at least 1 cm were considered undetermined, as conservative threshold based on the vertical distribution of artifacts. To identify potential post-depositional processes, artifact lengths were classed according to eight categories^107^ and artifact weights were separated into six classes based on their overall distribution. To identify potential artifact clustering and spatial correlations, the horizontal distribution of lithic artifacts from GH3 was analyzed using a χ^2^ test with a Monte Carlo simulation (nsim = 39)^108^. The type of distribution (aggregated, segregated, or random) and the corresponding radii were determined using Ripley’s K function. A Monte Carlo spatial scan test determined cluster locations. Relative spatial distributions and artifact cluster significances were assessed with the relative risk function. Artifact spatial correlations were evaluated with the nearest neighbor equality function^109,110^. Analyses were performed using the R spatstat package^111,112^.

### Black carbon identification

To evaluate past burning events, excavated sediments were analyzed for BC, the sum of organic fire residues from charcoal to soot^113^. In the environment, BC sedimentary input is driven by fire activity that is in turn, influenced by climate and/or human activity^60,114,115^.

At Româneşti, 45 samples were taken from each geological horizon except GH4 at 1 m horizontal intervals following the lithic artifact distributions across the eastern profile. Samples were dried (50 °C), sieved (< 2 mm) and milled. Organic carbon contents were determined with a Soli TOC Cube^116^. The quantity and quality of BC content was assessed by its oxidation to BCPA^117^ with modifications by Brodowski et al.^118^ and Kappenberg et al.. A threshold of 5 mg of organic carbon per sample was maintained and BC quantification was restricted to BPCAs with five and six carboxyl groups (B5CA and B6CA). BPCAs were measured using a Packard 6890 gas chromatograph equipped with a flame ionization detector and an HP-5 capillary column (30 m × 0.32 mm i.d., 0.25 mm film thickness; for oven program see Brodowski et al.^118^. BPCA yield was corrected for CO_2_ loss and insufficient conversion of BC to BPCAs using a factor of 2.27 to provide a conservative, minimum estimate of total BC in the sediment. The internal standard (citric acid) was recovered with a mean value of 73%.

The relative proportion of BPCA with 5–6 carboxyl groups depends on the combustion temperature. The hotter a fire burns, the more B6CA is formed, thus the ratio of B5CA to B6CA relates inversely to combustion temperature^45,120^.

### Raw materials and MAN analysis

Lithic artifact raw materials were petrographically characterized and sourced by comparison to geological samples derived from field surveys surrounding the site^46^. All piece-provenienced artifacts were macroscopically analyzed and assigned to an established raw material category and type. Lithic assemblages were then submitted to a MANA that grouped artifacts with similar macroscopic features and defined the number of MANs for each raw material category determined^121,122^.

A retouched obsidian blade from GH3 was analyzed with a NITON XL3t portable X-ray fluorescence (pXRF) reader for trace elements at three different locations for 160 s. Uncorrected results were averaged and compared to published elemental values for continental European sources^47,123,124^.

### Techno-typological analysis

The lithic analysis of the GH3 assemblage combined a *chaîne opératoire* approach with an attribute analysis^125–127^. The analysis used nearly all piece-provenienced artifacts from GH3 except those from unit P104 that were archived for future residue analyses.

Lithic artifacts were classified as cores, debitage, and undetermined artifacts. Core types were distinguished according to their reduction methods while debitage was subdivided into flakes (l≈w), blades (l > 2 × w with sub-parallel edges) and bladelets (blades where w < 12 mm)^129^. Undetermined artifacts were defined as those with no recognizable flaking axis or coherent set of negative scars.

Debitage was measured along the flaking axis and the preservation was defined as either complete, semi-complete (meso-proximal, meso-distal), or fragmented (proximal, mesial, or distal)^130^. Techno-typological analysis was conducted exclusively on complete and semi-complete debitage. Knapping platforms and bulb morphologies were classified according to Inizan et al.^131^ and Pelegrin^132^. Overhang abrasion was noted for cores and debitage. Debitage flaking angles (the intersection of the dorsal face with the platform) were identified as acute (< 70°), or moderately acute (70–89°). Artifacts preserving cortical surfaces were grouped as semi-cortical (< 50%), extensively cortical (51–90%), or entames (91–100%). Negative orientation was determined according to the artifact flaking direction. Blade and bladelet lateral edges were recorded as sub-parallel, convergent, or off-axis. Longitudinal profiles were sorted according to Bon^133^. Retouch type, position, and extent was described according to Inizan et al.^131^.

Debitage was additionally grouped into technological categories: cortical (entames and extensively cortical), blank (lithic artifacts flattening core convexities; i.e., blades, bladelets, and flakes), management (lithic artifacts renewing core convexities), tablets (lithic artifacts renewing the core platform), and burin spalls^125^. Tool typologies were classified according to Inizan et al.^131^.

### Lithic use-wear analysis

289 piece-provenienced lithic artifacts were selected from four units (N103–N106) for use-wear and microfracture analysis. Artifact selection was based on analytical suitability (i.e., raw material, size, edge preservation, burning, patination) independent of techno-typological classification ^134^. Additionally, 56 (semi-) complete Dufour bladelets and two manuports (all from GH3) were analyzed. Macro and micro use-wear analysis examined the entire perimeter of the dorsal and ventral sides of the artifacts. Use-wear traces were systematized in three main categories: abrasive (e.g., striations), impact (surface macro fractures), and micro polish (i.e., sheen). Macro analysis of artifact edges and surfaces (including fractures, edge scarring, and surface abrasion) was performed with a digital microscope (ZEISS SmartZoom 5, 1.6 × objective). Micro-polished areas were analyzed using a reflected light microscope (ZEISS Axio Scope.A1 MAT, objective EC Epiplan 10 × /0.25 M27 FWD = 11.0 mm and Objective EC Epiplan 20 × /0.4 M27 FWD = 3.2 mm). All photographs were acquired with the dedicated ZEISS Zen Core software employing the image Extended Depth of Focus (EDF) stacking module to generate in-focus images. Polished surfaces were qualitatively categorized and described using common terminology and categories ^135–140^.

Photographs (including overviews, areas of interest and particular macro features) were edited using GIMP (free open-source image editor, available at https://www.gimp.org/, v.2.10.18). All edited images were later combined and processed using Inkscape (free and open-source vector graphics editor, available at https://inkscape.org/, v.1.1).

## Supplementary Information

Supplementary Information 1.

Supplementary Information 2.

Supplementary Information 3.

## Data Availability

All data generated or analyzed during this study are included in this published article and its supplementary information files.
