# Supplementary material for: Aurignacian dynamics in Southeastern Europe based on spatial analysis, sediment geochemistry, raw materials, lithic analysis, and use-wear from Românești-Dumbrăvița
Source: Sci Rep. 2022 Aug 19;12:14152. doi: 10.1038/s41598-022-15544-5 (PMC9391429; doi:10.1038/s41598-022-15544-5)
Supplement: Supplementary file 1 — Supplementary Information 1. [file 41598_2022_15544_MOESM1_ESM.docx]

Supplementary Information for

**Aurignacian dynamics in Southeastern Europe based on spatial analysis, sediment geochemistry, raw materials, lithic analysis and use-wear from Românești-*Dumbrăvița***

Wei Chu^*^, Scott McLin, Luisa Wöstehoff, Alexandru Ciornei, Jacopo Gennai, João Marreiros, Adrian Doboș

Corresponding author: Wei Chu, email: w.chu@arch.leidenuniv.nl


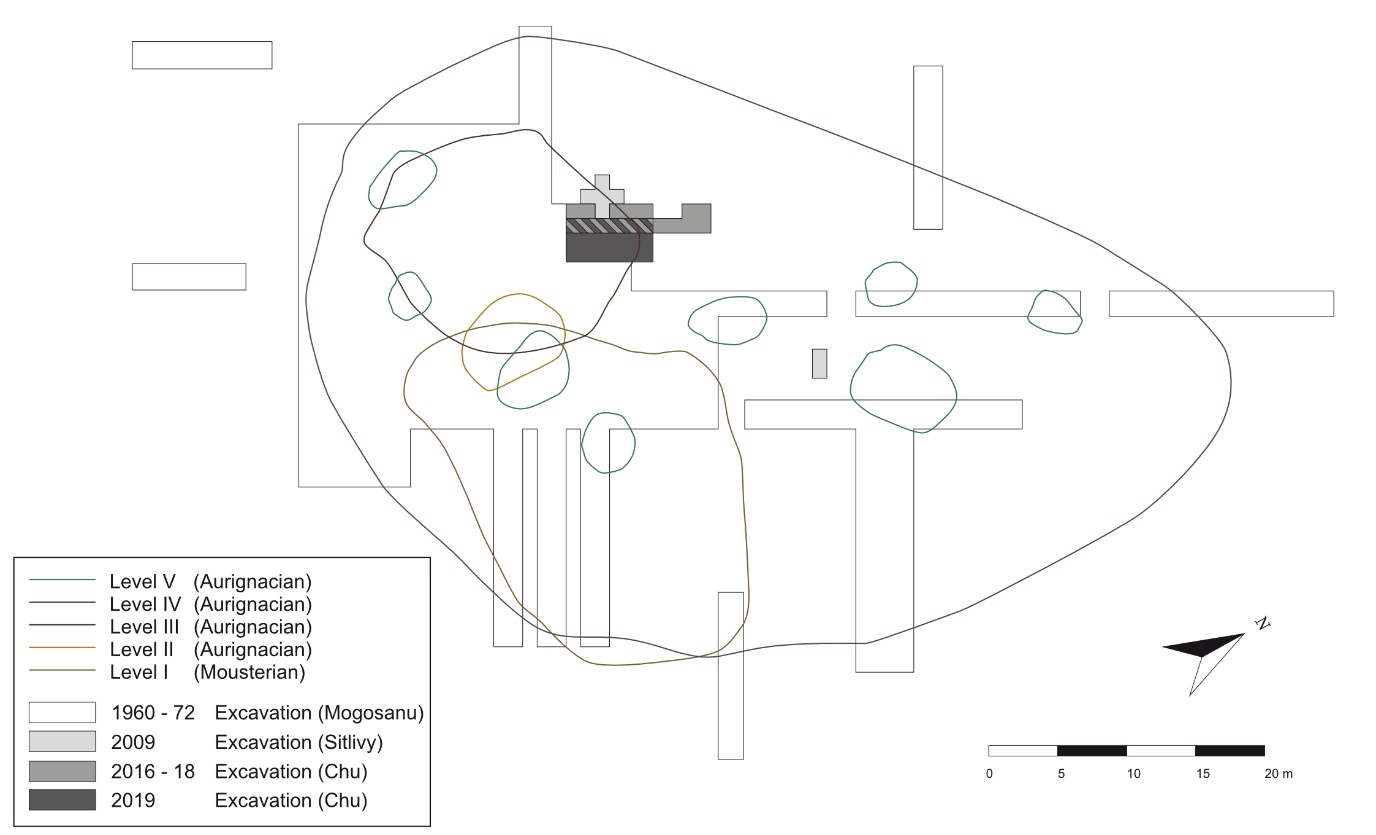


**Supplementary Figure 1.** Synthetic map of excavations at Românești-*Dumbrăvița* I. Outlines of artifact levels and earlier excavations are reported after Mogoșanu^1^ and the 2016-19 excavations are oriented according to the test-trenches from Sitlivy et al.^2,3^.

**
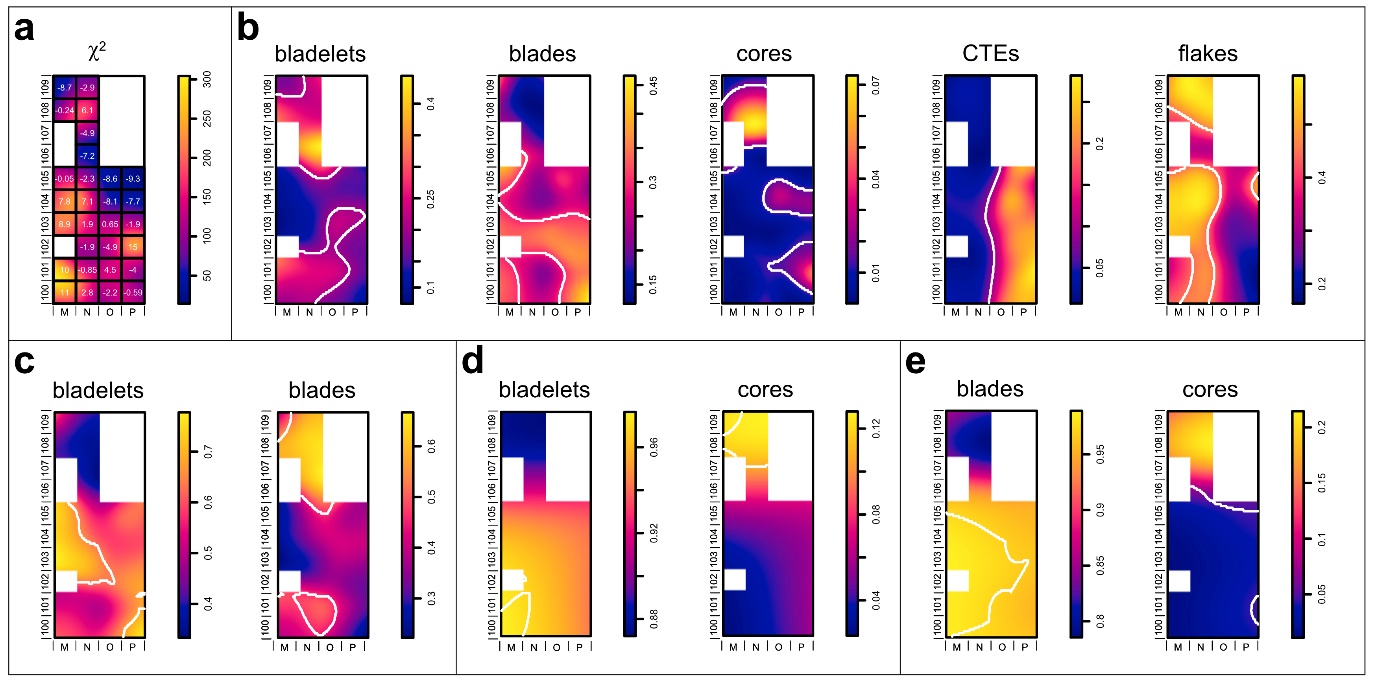
.**

**Supplementary Figure 2** Density plots of relative abundance of lithic types in GH3 using Scott’s rule of thumb for bandwidth selection for the kernel estimation^4^. (**a**) Chi squared test results showing Pearson’s residual; (**b**) relative risk function of lithic artifact types (**c**) relative risk function of blades – bladelets distributions; (**d**) relative risk function of bladelets – cores distributions; (**e**) relative risk function of blades – cores distributions. White lines show contours of significant occurrences of lithic types. Scales are estimated pieces per kernel density surface (p/kds).


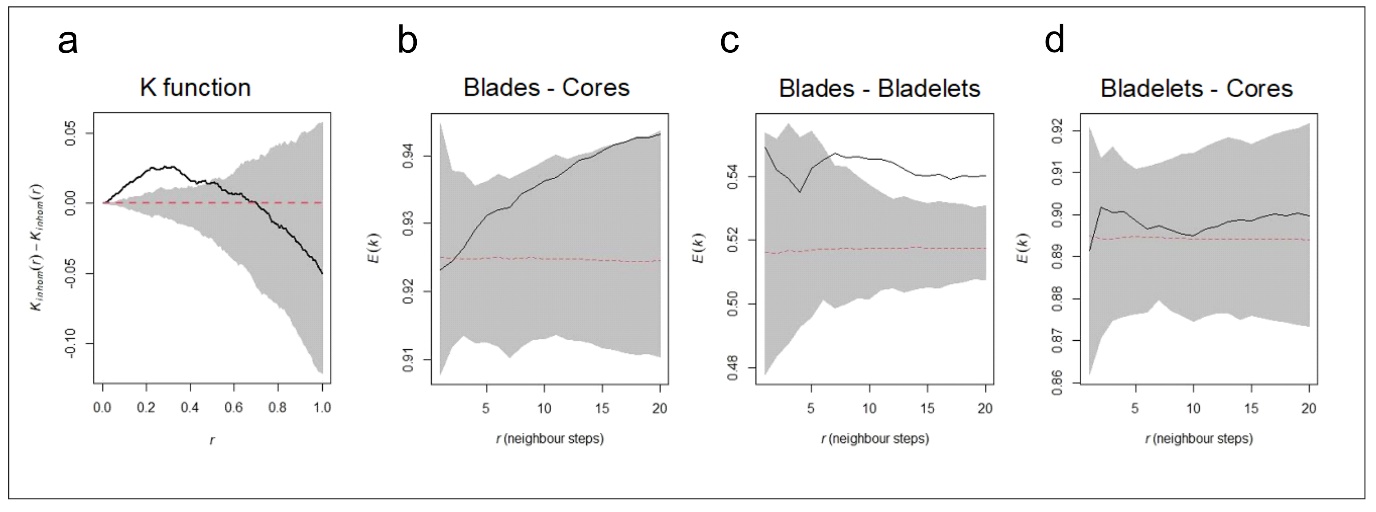


**Supplementary Figure 3** (**a**) Results from the Ripley’s K function; (**b-d**) results from the nearest neighbor equality function. Black line indicates observed value; red dashed line indicates the theoretical value; the Monte Carlo envelope (gray) indicates within where random distribution can be expected. If the observed value falls above the envelope, the correlation is positive, and artifacts of the same type tend to be closer than to artifacts of the other type. If the observed value falls below the envelope, the correlation is negative, and artifacts of the same type tend to repel each other. There is no correlation between the different artifact types when the observed pattern falls within the envelope


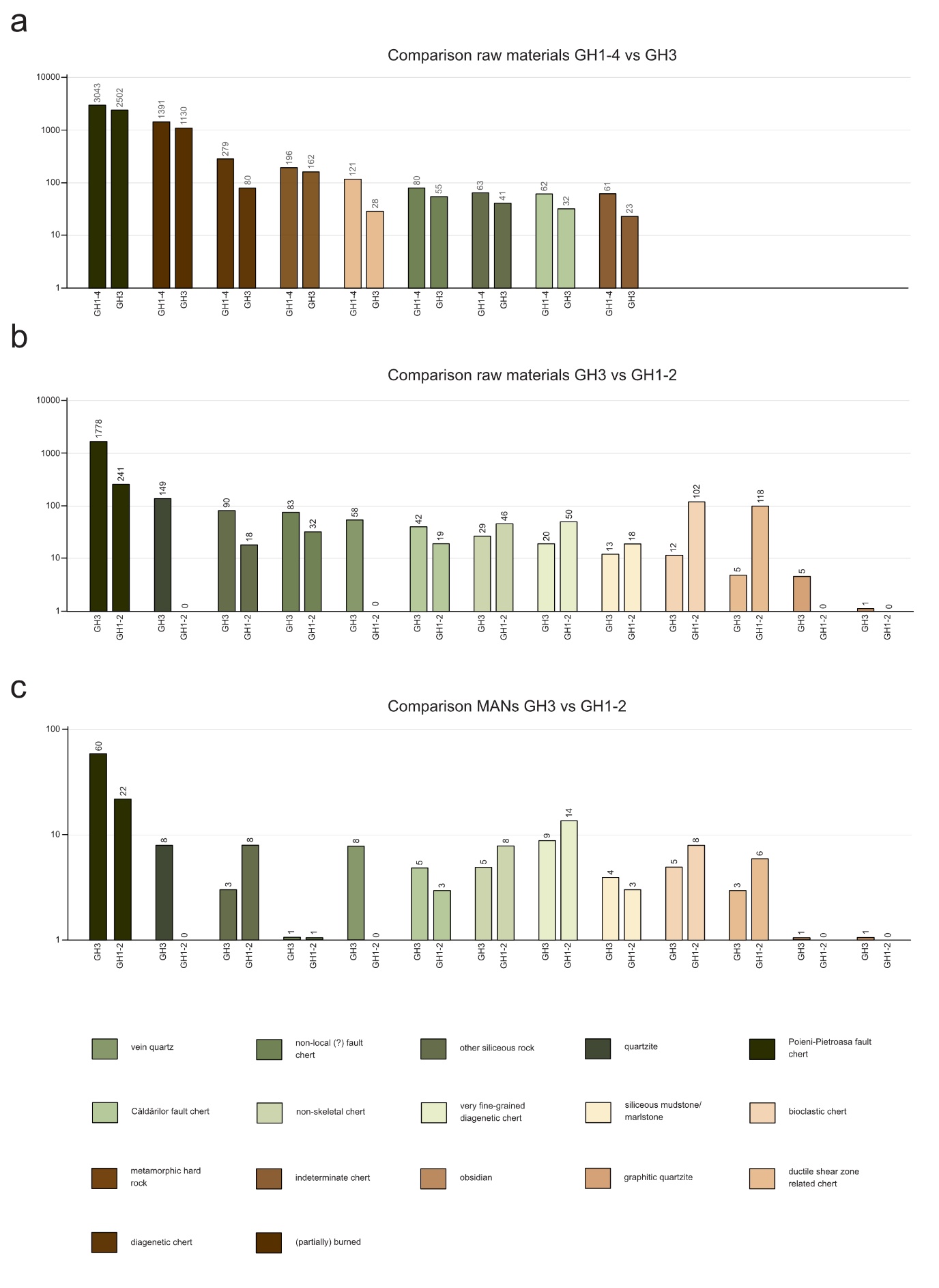


**Supplementary Figure 4** Comparisons of (**a**) knapped lithic artifact counts by raw material from Românești-*Dumbrăviţa* I, GH1-4 and GH3; (**b**) lithic artifact counts by raw material from Românești-*Dumbrăviţa* I, GH3 and GH1-2; (**c**) lithic counts by MANs from Românești-*Dumbrăviţa* I, GH3 and GH1-2.

**
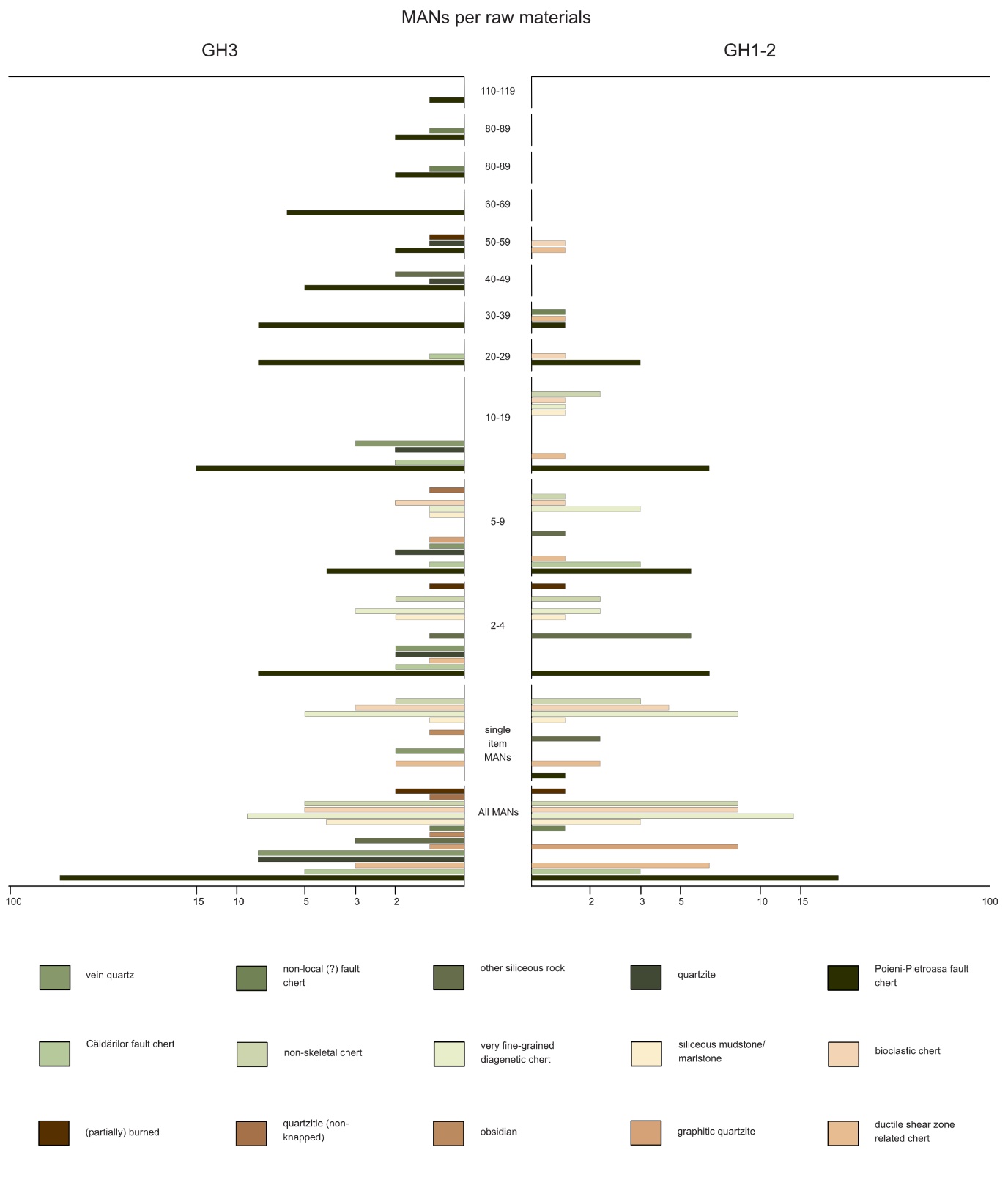
**

**Supplementary Figure 5** Counts of MANs by raw materials from Românești-*Dumbrăviţa* I, GH3 and GH1-2.

**
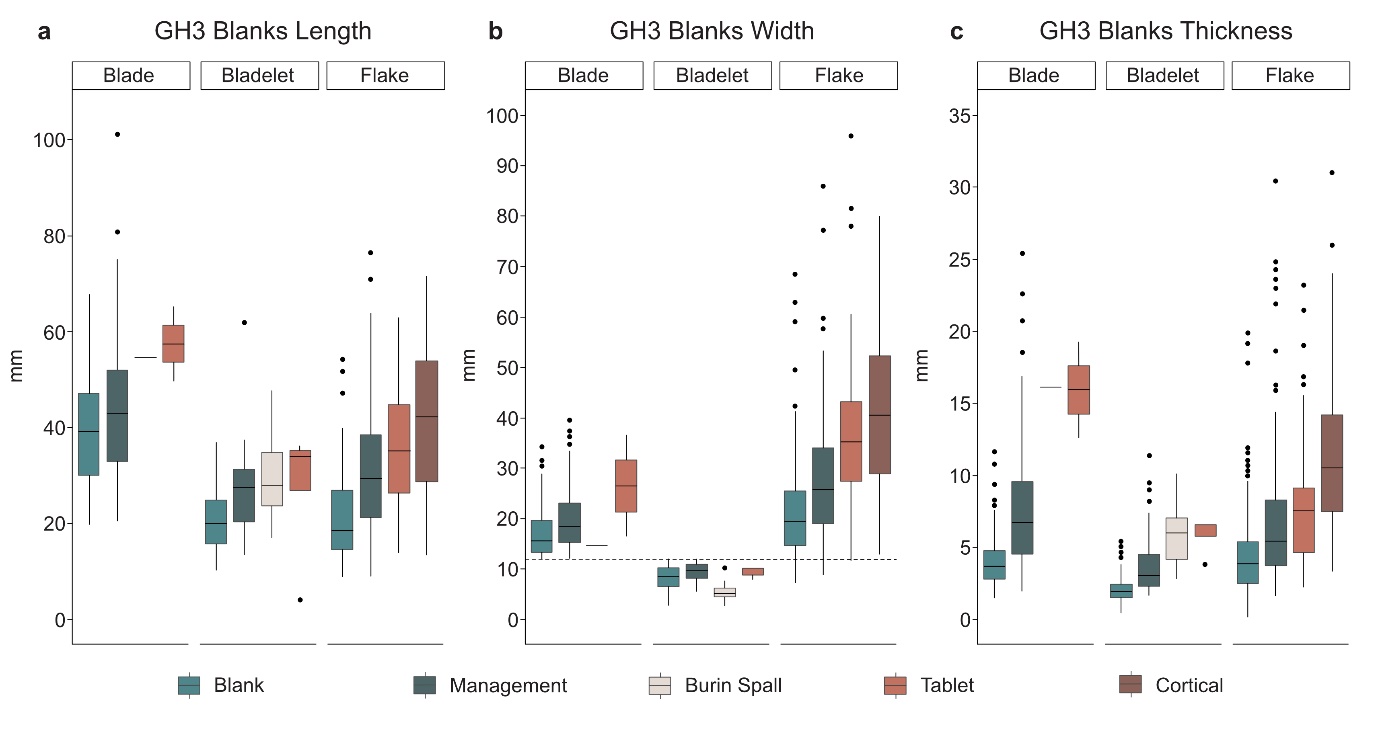
**

**Supplementary Figure 6** Blank dimensions from Românești-*Dumbrăviţa* I, GH3. (**a**) Length was determined with complete blanks only while (**b**) width and (**c**) thickness were determined with complete and semi-complete blanks. Dashed line represents the 12 mm threshold between blades and bladelets.

**
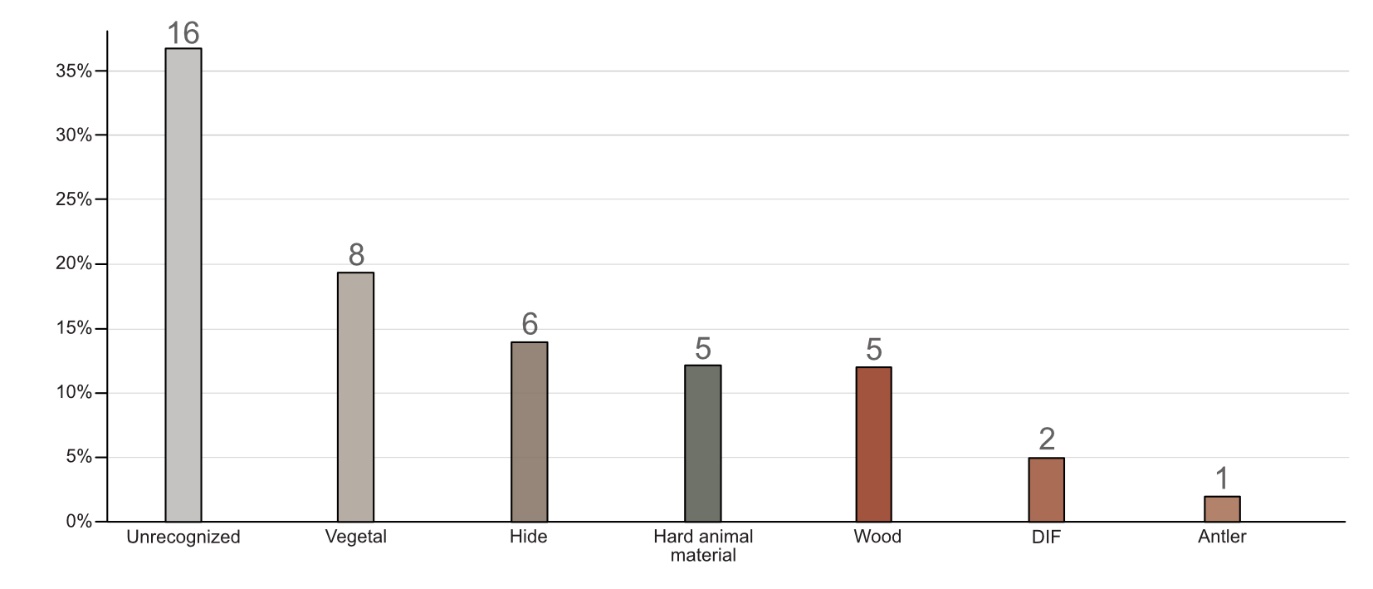
**

**Supplementary Figure 7** Column chart of the types of use-wear traces found on lithic artifacts from Românești-*Dumbrăviţa* I (N=43).

**
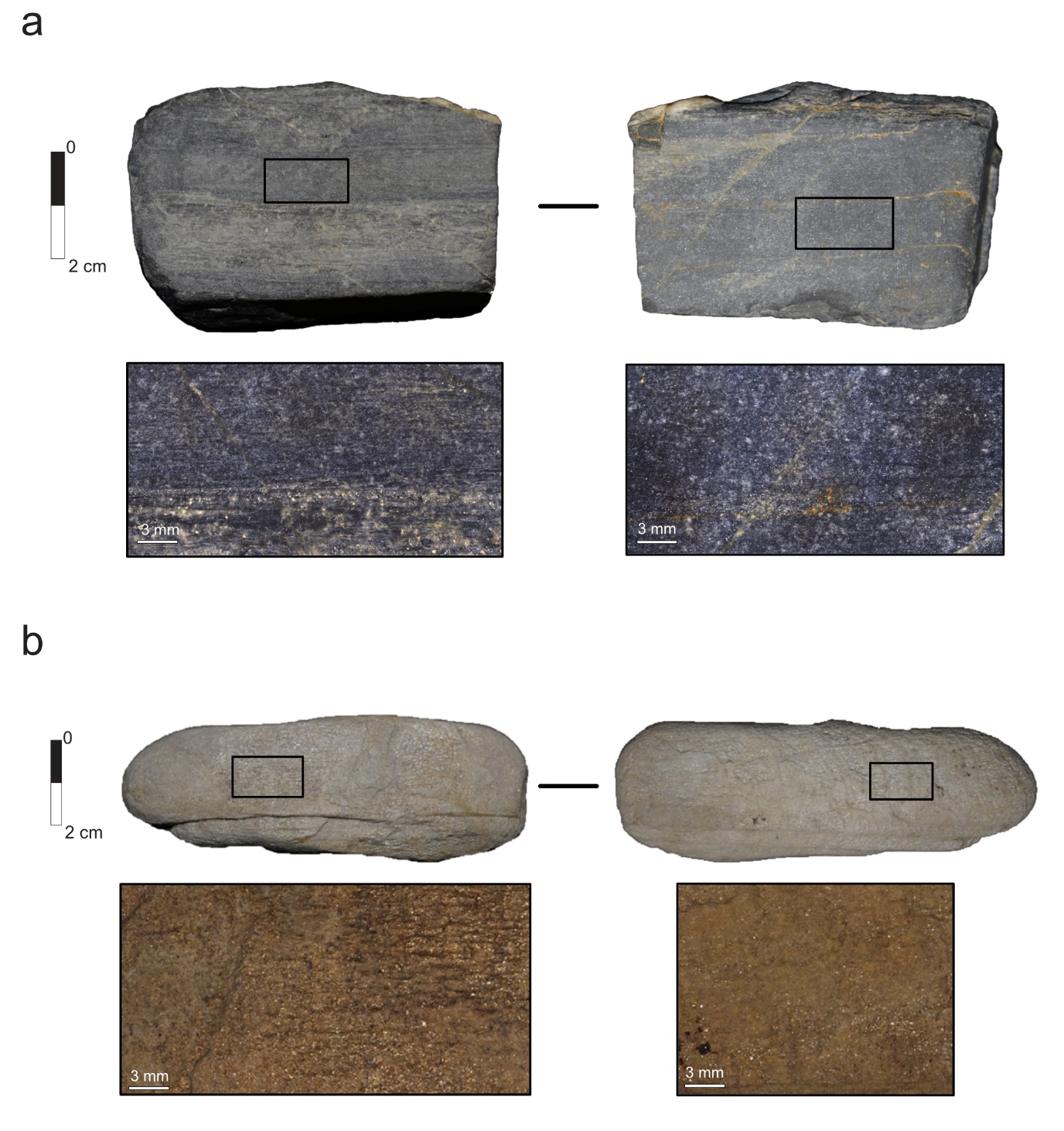
**

**Supplementary Figure 8.** Use-wear traces found on the two manuports (groundstones) from Românești-*Dumbrăvița* I, GH3 (**a**) Manuport and micrographs of a concave grove running along the longitudinal axis of the manuport (on the right, showing smother surface texture, including longitudinal striations) and (on the left) the opposite unused surface for comparison; (**b**) manuport and micrographs of both surfaces of the tool. On the right at the lowest point of the concave groove running through the center of the longest axis and the opposite side, where no use-wear has been found for comparison. The surfaces interpreted as used on both manuports show smoother surface texture, including longitudinal striations, when compared with the opposite surface.

# **Supplementary References**

1. Mogoșanu, F. *Paleoliticul din Banat*. vol. 32 (Editura Academiei Republicii Socialiste Romānia,
    1978).
2. Sitlivy, V. *et al.* The earliest Aurignacian in Romania: new investigations at the open air site of
    Româneşti-Dumbrăviţa I (Banat). *Quartär* **59**, 85–130 (2012).
3. Sitlivy, V. *et al.* Placing the Aurignacian from Banat (southwestern Romania) into the European
    Early Upper Paleolithic Context. in *Modes de contactes et de deplacements au Paléolithique
    Eurasiatique* (eds. Otte, M. & Brun-Ricalens, F. L.) vol. 8 243–277 (ERAUL, 2014).
4. Scott, D. W. Theory, Practice and Visualization. *NY John Wiley Sons* (1992).
